# Bacteria and Archaea Regulate Particulate Organic Matter Export in Suspended and Sinking Marine Particle Fractions

**DOI:** 10.1128/msphere.00420-22

**Published:** 2023-04-24

**Authors:** Choaro D. Dithugoe, Oliver K. I. Bezuidt, Emma L. Cavan, William P. Froneman, Sandy J. Thomalla, Thulani P. Makhalanyane

**Affiliations:** a Southern Ocean Carbon-Climate Observatory (SOCCO), Council of Scientific & Industrial Research (CSIR), Rosebank, Cape Town, South Africa; b SARChI Chair: Marine Ecosystems and Resources, Department of Entomology & Zoology, Rhodes University (RU), Makhanda, Eastern Cape, South Africa; c SARChI Chair: Marine Microbiomics, microbiome@UP, Department of Biochemistry, Genetics and Microbiology, University of Pretoria (UP), Hatfield, Pretoria, South Africa; d Imperial College London, Berks, Silwood Park, Berkshire, United Kingdom; Clemson University Department of Biological Sciences

**Keywords:** Southern Ocean, carbon export, functional capacity, marine fractions, Marine Snow Catcher, metagenomics, particulate organic matter, prokaryotes

## Abstract

The biological carbon pump (BCP) in the Southern Ocean is driven by phytoplankton productivity and is a significant organic matter sink. However, the role of particle-attached (PA) and free-living (FL) prokaryotes (bacteria and archaea) and their diversity in influencing the efficiency of the BCP is still unclear. To investigate this, we analyzed the metagenomes linked to suspended and sinking marine particles from the Sub-Antarctic Southern Ocean Time Series (SOTS) by deploying a Marine Snow Catcher (MSC), obtaining suspended and sinking particulate material, determining organic carbon and nitrogen flux, and constructing metagenome-assembled genomes (MAGs). The suspended and sinking particle-pools were dominated by bacteria with the potential to degrade organic carbon. Bacterial communities associated with the sinking fraction had more genes related to the degradation of complex organic carbon than those in the suspended fraction. Archaea had the potential to drive nitrogen metabolism via nitrite and ammonia oxidation, altering organic nitrogen concentration. The data revealed several pathways for chemoautotrophy and the secretion of recalcitrant dissolved organic carbon (RDOC) from CO_2_, with bacteria and archaea potentially sequestering particulate organic matter (POM) via the production of RDOC. These findings provide insights into the diversity and function of prokaryotes in suspended and sinking particles and their role in organic carbon/nitrogen export in the Southern Ocean.

**IMPORTANCE** The biological carbon pump is crucial for the export of particulate organic matter in the ocean. Recent studies on marine microbes have shown the profound influence of bacteria and archaea as regulators of particulate organic matter export. Yet, despite the importance of the Southern Ocean as a carbon sink, we lack comparable insights regarding microbial contributions. This study provides the first insights regarding prokaryotic contributions to particulate organic matter export in the Southern Ocean. We reveal evidence that prokaryotic communities in suspended and sinking particle fractions harbor widespread genomic potential for mediating particulate organic matter export. The results substantially enhance our understanding of the role played by microorganisms in regulating particulate organic matter export in suspended and sinking marine fractions in the Southern Ocean.

## INTRODUCTION

The Southern Ocean plays a significant role in carbon cycling, buffering the impacts of climate change by accounting for 50% of the total oceanic uptake of CO_2_ (1–3). Phytoplankton primary production and carbon export to the deep ocean (i.e., the biological carbon pump [BCP]) (4, 5), are considered a major contributor to the sink of natural CO_2_, removing approximately 33% of the global organic carbon flux as particulate organic carbon (POC) (6, 7). However, only a small fraction of the organic carbon fixed by phytoplankton in surface waters ultimately reaches the ocean interior (8, 9). The factors that control the fraction of production, which is exported, or how effectively this material is transferred to depth remain unclear. Factors that regulate phytoplankton growth, particle formation, rates of sinking, and remineralization all modify the extent to which fixed POC is effectively exported and transformed into dissolved organic carbon (DOC) and CO_2_. Altogether, these factors determine the efficiency of the BCP (10, 11).

Particulate organic matter (POM), derived from phytoplankton, in addition to detritus and zooplankton fecal pellets, typically aggregates at the surface and may be separated into suspended and sinking particles based on their sinking velocity (12–14). The particle composition of the suspended and sinking fractions is highly variable in time and space, with different efficiencies in POM export and attenuation (4, 15). Earlier studies focused on the role of sinking particles and their importance in enhancing POM export (16). Recently, the suspended particles have also been observed in mesopelagic sediment traps (17), suggesting that they play a large role in exporting carbon to depth by physical processes such as the particle injection pumps (e.g., ocean mixing and migrant pump) (18, 19). In addition, the suspended particles are produced at depth from degradation of large sinking particles (20). Therefore, both particle types (suspended and sinking) are important for determining the role of the BCP in exporting POM to depth (14, 17, 21, 22).

Marine prokaryotes (bacteria and archaea), in particular those associated with suspended and sinking particles, have been shown to mediate key processes linked to the BCP (23–26). Prokaryotic diversity influences the composition of DOC, which includes a diverse range of molecules, which may be biologically labile (e.g., amino acids and glucose). These molecules are rapidly remineralized by microbes in the surface ocean to produce dissolved inorganic carbon (DIC), thus reducing export efficiencies (the microbial loop) (24, 27). Alternatively, prokaryotic-produced recalcitrant dissolved organic carbon (RDOC) (e.g., lignin and lipids) may be exported to the deep ocean, facilitating longer-term storage (28–30). A small percentage of prokaryotic DOC production is refractory (rDOC) (e.g., ~5 to 7% derived from glucose), which resists rapid remineralization and further degradation (31–34). The rDOM produced by prokaryotic degradation of complex organic carbon accumulates in the ocean interior, accounting for >95% of the large DOC pool (35, 36). This long-lived reservoir plays an important role in shaping global climate by sequestering CO_2_ from the atmosphere (37). Prokaryotic production of RDOC involves complex compounds that are difficult to manage, while rDOC represents labile compounds that are resistant to further degradation; both of these components of DOC form part of the microbial carbon pump (MCP) (34). The MCP sequestrates organic carbon by aiding the transfer of DOC to the deep ocean (38–40). Indeed, in instances where the microbial loop dominates (i.e., a system with small-celled, nonsinking particles and low POC flux) the MCP can be considered the prevailing mechanism for carbon sequestration (37). Despite the intricate role of prokaryote diversity and activity in regulating both the BCP and MCP, we lack genomic information regarding the phylogeny and function of prokaryotes linked with suspended and sinking particle-pools in the ocean.

The composition of the suspended and sinking particle pool (either labile, semi-labile, semi-recalcitrant, or recalcitrant) (41) may also determine the change in phylogenetic and genomic potential of the prokaryotic community (42, 43). Prokaryotes associated with suspended and sinking particles utilize and/or change in response to labile and semi-labile POM (44), in addition to having the genomic potential to slowly degrade complex high-molecular-weight organic compounds such as carbohydrates or lipids from phytoplankton cells (45). As such, genomic potential differentiation is expected in prokaryotic distribution based on their ability to degrade particulate organic matter (POM) (46) in either the suspended or the sinking particle pool. In addition, the prokaryotic community can both attach and detach from particles, thus having the capacity to enrich the surrounding water with free-living (FL) prokaryotes with the genomic potential to degrade complex material (42).

**FIG 1:**
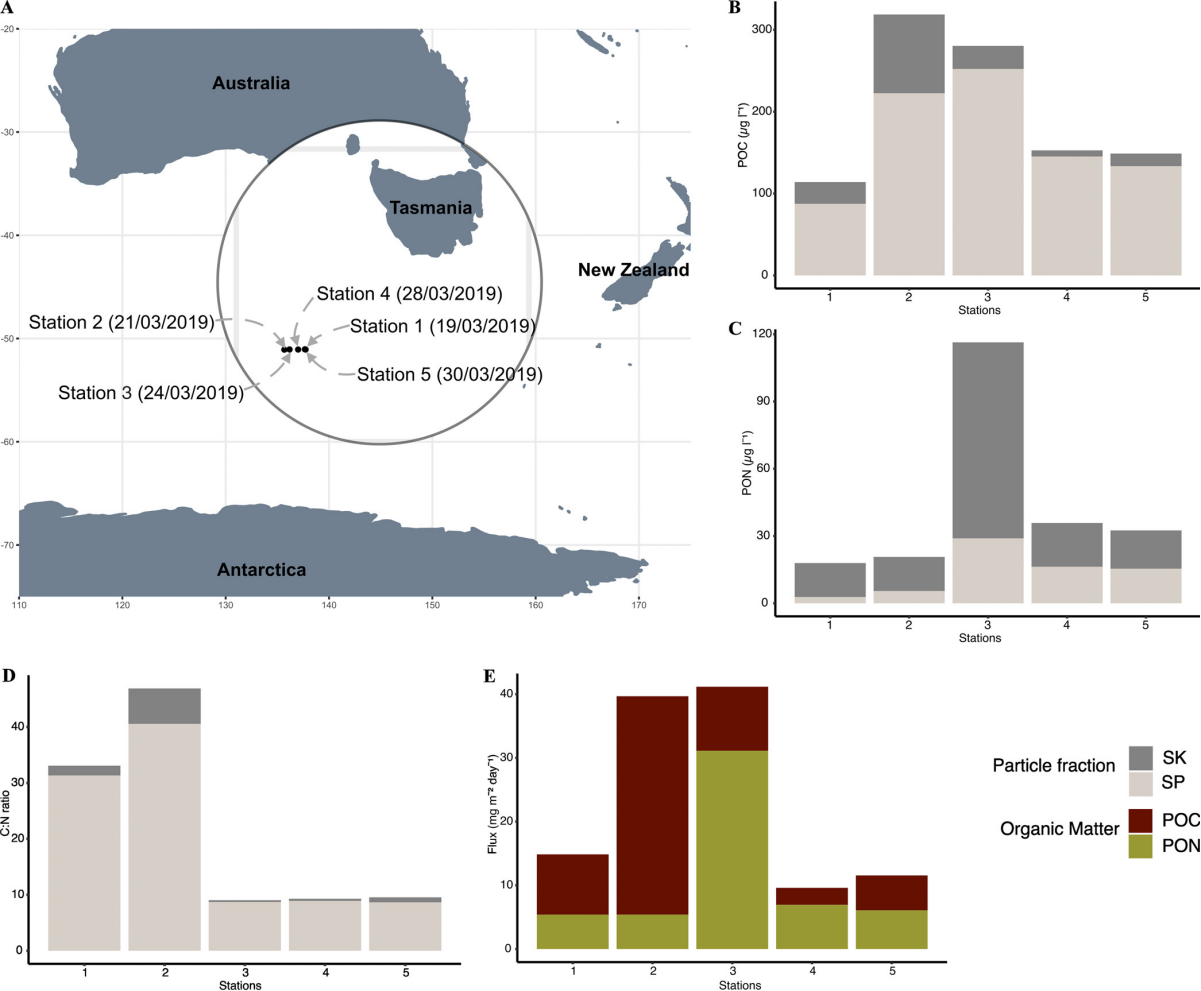
Marine Snow Catcher (MSC) deployment at Southern Ocean Time Series sites during the IN2019_V02 using RV *Investigator* collecting POC and PON from the suspended (SP) and sinking (SK) particle pool. (A) The sampling locations at the SOTS site stations 1 and 5 are slightly different in latitude and longitude (see Data Set S1, Tab 1). (B) POC concentrations for SP (light gray bars) and SK (dark gray bars); (C) PON concentration for SP and SK; (D) POC/PON ratio for SP and SK; (E) POC and PON export flux at five stations.

Here, we present the first assessment of prokaryotic genomic potential in suspended and sinking marine particle fractions collected with a Marine Snow Catcher (MSC) at five stations from the Southern Ocean Time Series (SOTS) site in the sub-Antarctic zone (SAZ) during austral autumn (Fig. 1). In addition to determining organic carbon and nitrogen flux, we specifically elucidate carbon and nitrogen cycling metabolic pathways linked to prokaryotes collected from the suspended and sinking particle fractions. By using metagenome-assembled genomes (MAGs) to link bacterial and archaeal genomes to POM sequestration, we provide insights into the role of prokaryotic community in organic matter export in the Southern Ocean.

10.1128/msphere.00420-22.6 DATA SET S1 (Tab 1) Detailed sampling of the Marine Snow Catcher from five Southern Ocean Time Series (SOTS) stations, including the date, time, and depth where the sample was collected. Sampling stations are numbered based on sampling date and time. (Tab 2) Predicted functional genes from unbinned bacterial contigs with gene description, module/pathways, and header. Each unbinned contig has a number that represent gene counts or copies. Gene counts greater than 1 are colored pink, while the gene counts equal to 0 are colored white. (Tab 3) Predicted functional genes from unbinned archaea contigs with gene description, module/pathways, and header. Each unbinned contig has a number that represents gene counts or copies. Gene counts greater than 1 are colored pink, while gene counts equal to 0 are colored white. (Tab 4) Classification of reconstructed MAGs from GTDB-Tk with completeness, contamination percentages, GC content, genome size, and number of rRNA genes. (Tab 5) Average nucleotide score of the reconstructed MAGs against RefSeq obtained from NCBI. A score above 95% represents similar taxa. (Tab 6) Predicted functional genes from reconstructed bacterial MAGs with gene description, module/pathways, and header. Each MAG has a number that represent gene counts or copies. Gene counts greater than 1 are colored pink, while gene counts equal to 0 are colored white. (Tab 7) Predicted functional genes from reconstructed archaeal MAGs with gene description, module/pathways, and header. Each MAG has a number that represent gene counts or copies. Gene counts greater than 1 are colored pink, while gene counts equal to 0 are colored white. Download Data Set S1, XLSX file, 0.1 MB. Copyright © 2023 Dithugoe et al. 2023 Dithugoe et al. https://creativecommons.org/licenses/by/4.0/ This content is distributed under the terms of the Creative Commons Attribution 4.0 International license.

## RESULTS

### Taxonomic profiling of raw reads.

**FIG 2:**
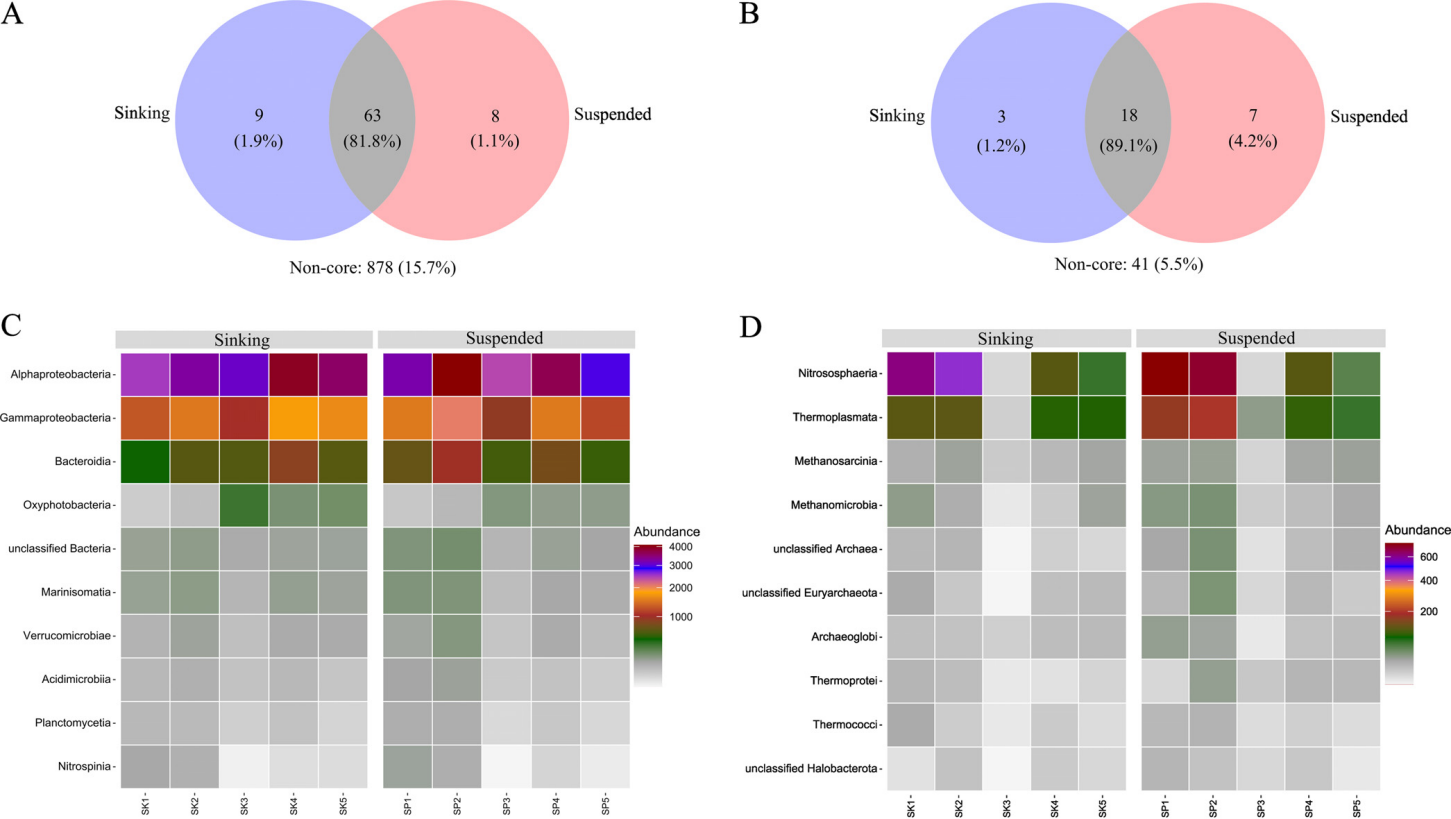
Taxonomic composition and distribution of the Southern Ocean Time Series prokaryotic communities, determined using single-copy marker genes (ribosomal protein genes) with the SingleM pipeline. (A) Venn diagram showing the core OTUs shared by suspended and sinking bacterial taxonomic composition; (B) Venn diagram showing the core OTU percentage of read abundance shared by suspended and sinking archaeal taxonomic composition; (C) heat map showing the percentages of abundance of the bacterial class composition in the suspended and sinking fractions at each station. Taxa with low abundance are colored blue, and those in higher abundance are white, while the highest are red. (D) Heat map showing the percentages of abundance of the archaeal class in the suspended and sinking communities at each station.

To elucidate bacterial and archaeal communities in suspended and sinking particle fractions, we assigned taxonomy to raw metagenomic reads. The operational taxonomic units (OTUs) within the top frequency cutoff of 80% were regarded as core OTUs, while those below 80% were excluded from further analysis as non-core OTUs. Additionally, core OTUs, with abundances below 0.1%, were excluded from downstream analysis. Bacterial communities shared 81.8% OTUs, while approximately 1.1% (suspended) and 1.9% (sinking) were unique, and 15.7% were non-core OTUs (Fig. 2A). Similarly, the archaea shared 89.1% OTUs, while ~4.2% (suspended) and about 1.2% (sinking) were unique, and 5.5% were non-core OTUs (Fig. 2B). Classification at the class level revealed that bacterial communities were dominated by *Alphaproteobacteria*, *Gammaproteobacteria*, and *Bacteroidia* in all stations for both the sinking and suspended fractions (Fig. 2C). Additionally, *Pelagibacteraceae* (*Alphaproteobacteria*) and *Flavobacteriaceae* (*Bacteroidia*), at the family level, were the most dominant taxa at all station in both suspended and sinking particle-pools (see Fig. S2A in the supplemental material). Very little difference was observed in bacterial community distribution when comparing the suspended and sinking particle-pools across all stations at the class level. However, at the family level, bacterial communities were diverse, with *Nitrospinaceae* (*Nitrospina*) only represented at station 1 and *Cyanobiaceae* (*Oxyphotobacteria*) only found at stations 3, 4, and 5. Interstation differences, at both class and family levels, were more apparent in the distribution of archaeal lineages (Fig. 2D; Fig. S2B). In general, archaeal communities were less dominant than bacteria at all stations. We observed *Nitrososphaeria* (*Nitrosopumilaceae*), *Thermoplasmata* (*Thermoplasmata* and *Thermoplasmataceae*), and *Methanosarcinia* (unclassified *Methanosarcinia*) in the suspended and sinking fractions (Fig. 2D; Fig. S2B). Stations 1 and 2 were dominated by *Nitrosophaeria*, *Thermoplasmata*, and *Methanomicrobia*. The station 3 suspended fraction was dominated by *Thermoplasmata*, *Thermoprotei*, and *Methanomicrobia*, while the sinking fraction was dominated by *Methanosarcinia*, *Archaeoglobi*, and *Thermoplasmata*. Stations 4 and 5 were also dominated by members of *Nitrosophaeria*, *Thermoplasmata*, and *Methanosarcinia*. Similar to bacterial communities, very few differences were observed when comparing archaea in the suspended and sinking particle-pools, at both class and family levels, with the exception of samples from station 3. The archaeal community at station 3 showed the most diverse composition and displayed the largest difference between suspended and sinking fractions.

10.1128/msphere.00420-22.2 FIG S1 Ancillary data from the Southern Ocean Time Series sites obtained from CTD data. (A) Temperature (degree Celsius) and salinity plot showing the five sampling stations. The colored circles represent the depths at which the samples were collected. (B) Chlorophyll (milligrams per cubic meter) depth profile for five stations determined by CTD fluorescence sensor and (C) the MLD integrated chlorophyll (milligrams per square meter). Download FIG S1, TIF file, 1.1 MB. Copyright © 2023 Dithugoe et al. 2023 Dithugoe et al. https://creativecommons.org/licenses/by/4.0/ This content is distributed under the terms of the Creative Commons Attribution 4.0 International license.

10.1128/msphere.00420-22.3 FIG S2 Overview of the prokaryotic community composition for five stations at the class and family level for the SP and SK particle-pool. The color scale represents read abundance. (A) Bacterial community composition; (B) archaeal community composition at both the class and family levels. Download FIG S2, TIF file, 1.9 MB. Copyright © 2023 Dithugoe et al. 2023 Dithugoe et al. https://creativecommons.org/licenses/by/4.0/ This content is distributed under the terms of the Creative Commons Attribution 4.0 International license.

### Functional annotation of unbinned metagenomic contigs.

**FIG 3:**
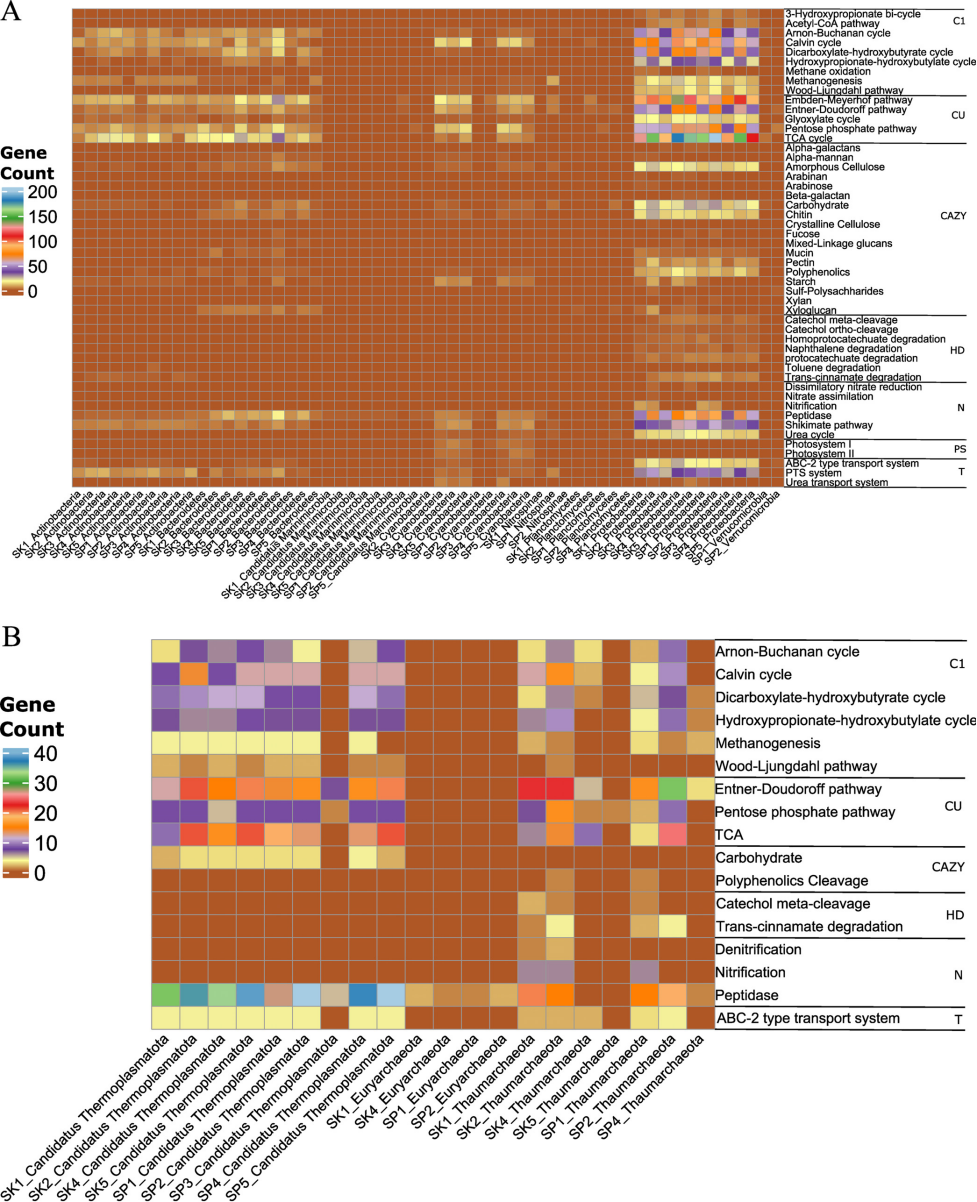
Predicted genes for both complex, central, and chemoautotrophic prokaryotic community from unbinned contigs based on the DRAM tool in both the suspended (SP) and sinking (SK) particle pool. The predicted genes are involved in chemoautotrophs (C1), carbon utilization (CU), carbohydrate active enzymes (CAZymes [CAZY]), hydrocarbon degradation (HD), nitrogen metabolism (N), photosynthesis (PS), and transporter systems (T). The color scale represents predicted gene counts, including gene copies. Detailed gene names and copies are given in Data Set S1: Tab 2 for bacteria and Tab 3 for archaea. (A) Bacterial community functional annotation; (B) archaeal community functional annotation.

*Proteobacteria*, the most dominant bacterial phyla (see Text S1 in the supplemental material), were predicted to possess more genes with genomic potential for degrading high-molecular weight organic compounds (CAZymes and hydrocarbon degradation) (Fig. 3A). Several bacteria, recovered from all stations, including members of the *Actinobacteria*, had more genes involved in the degradation of carbohydrates (acetyl xylan esterase), amorphous cellulose (β-glucosidase), and chitin (lysozyme type G), with more gene copies in the sinking fraction—mostly at stations 1 and 2 (see Data Set S1, Tab 2, in the supplemental material). Interestingly, only archaea from the class *Thaumarchaeota* and “*Candidatus* Thermoplasmatota” had more gene copies involved in the degradation of high-molecular-weight compounds such as carbohydrates (esterases), polyphenolics (laccase/*p*-diphenol:oxygen oxidoreductase), catechol (4-oxalocrotonate tautomerase), and *trans*-cinnamate (3-phenylpropionate/cinnamic acid dioxygenase). These archaea were found mostly in samples retrieved from suspended and sinking fractions of stations 1 and 2, respectively (Fig. 3B; Data Set S1, Tab 3).

10.1128/msphere.00420-22.1 TEXT S1 Detailed methodology and results section. The section includes additional data collected from a conductivity, temperature, and depth (CTD) instrument and the Marine Snow Catcher (MSC). The methods include further information regarding sample site descriptions, MSC deployment, and the sequencing protocols. The supplemental results section outlines the analyzed data from ancillary data, including water mass, phytoplankton biomass, and the particulate organic carbon (PON) and particulate organic nitrogen (PON) concentrations. This section also includes details regarding the taxonomic profile and genomic potential of unbinned metagenomic contigs, as well as the reconstructed metagenome-assembled genomes (MAGs). Download Text S1, DOCX file, 0.03 MB. Copyright © 2023 Dithugoe et al. 2023 Dithugoe et al. https://creativecommons.org/licenses/by/4.0/ This content is distributed under the terms of the Creative Commons Attribution 4.0 International license.

Archaea had a higher diversity of genes implicated in organic nitrogen degradation (e.g., protein degradation to amino acids) than bacteria. These genes include amino-, carboxy-, endo-, exo-, and isopeptidases, and most were ascribed to bacteria in suspended fractions (Data Set S1, Tab 3). Bacteria had more aminopeptidase and carboxypeptidase genes, mostly in the sinking fraction, and endopeptidase genes were found mainly in the suspended fraction (Data Set S1, Tab 2). On the other hand, archaea in stations 1 (both fractions) and 2 (sinking fraction) had genes involved in nitrification (ammonia monooxygenase). In addition to these stations, samples from the sinking fraction had several genes involved in denitrification (nitrite reductase). We identified genes implicated in the storage of nitrogen from bacterial taxa. These genes include those involved in the Shikimate pathway (3-deoxy-7-phosphoheptulonate synthase, chorismite synthase, and shikimate kinase) and urea cycle (argininosuccinate lyase and synthase). In general, we found more metabolic genes in the sinking fraction (Fig. 3A).

Moreover, bacterial and archaeal contigs from all stations were predicted to have genes involved in CO_2_ fixation and to produce RDOC via chemoautotrophic pathways (Text S1). These chemoautotrophic pathways, and related gene copies, were higher at stations 1 and 2 (suspended), station 3 (both suspended and sinking), and stations 4 and 5 (sinking). Our analyses suggest that bacteria harbored more genes or chemoautotroph-related pathways than archaea. In addition, these bacteria had several transporter systems, including ABC-2 type (ABC-2.A/P), phosphoenolpyruvate(PEP):carbohydrate phosphotransferase (PTS; PTS-Gut-EIIA) and urea (urtA-E) transport systems in both size fractions. In contrast, archaea had genes involved in only the ABC-2 type (ABC-2.A/P) transport system, mostly in the sinking fraction.

### Functional profiling of bacterial and archaeal genomes from the suspended and sinking particle-pools.

**FIG 4:**
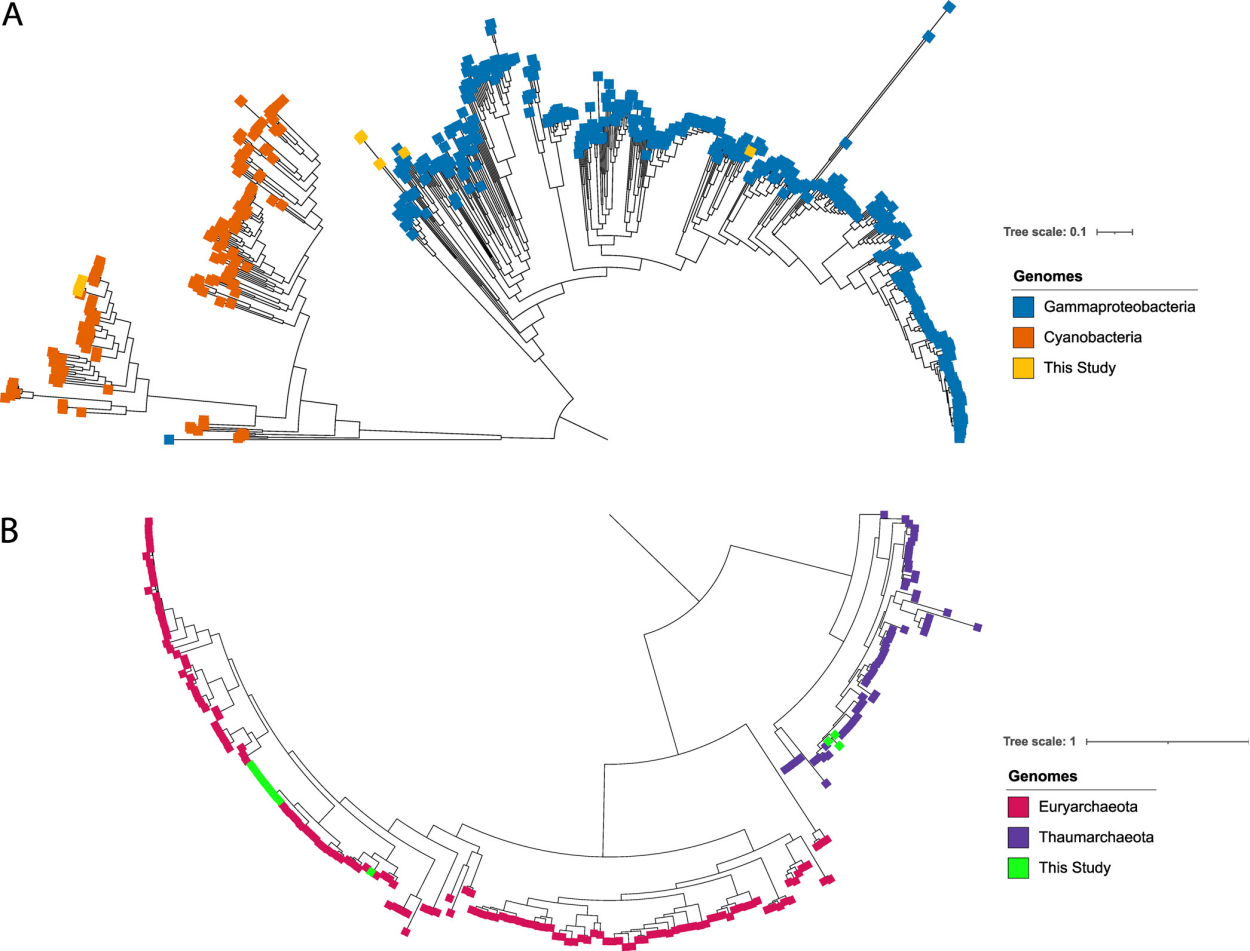
Phylogenomic inference of our 24 MAGs. The phylogenomic tree was based on alignment of 40% of the marker gene present in our MAGs. (A) Bacterial MAGs (yellow) against *Gammaproteobacteria* (blue) and *Cyanobacteria* (orange); (B) archaeal MAGs (green) against *Euryarchaeota* (red) and *Thaumarchaeota* (purple).

**FIG 5:**
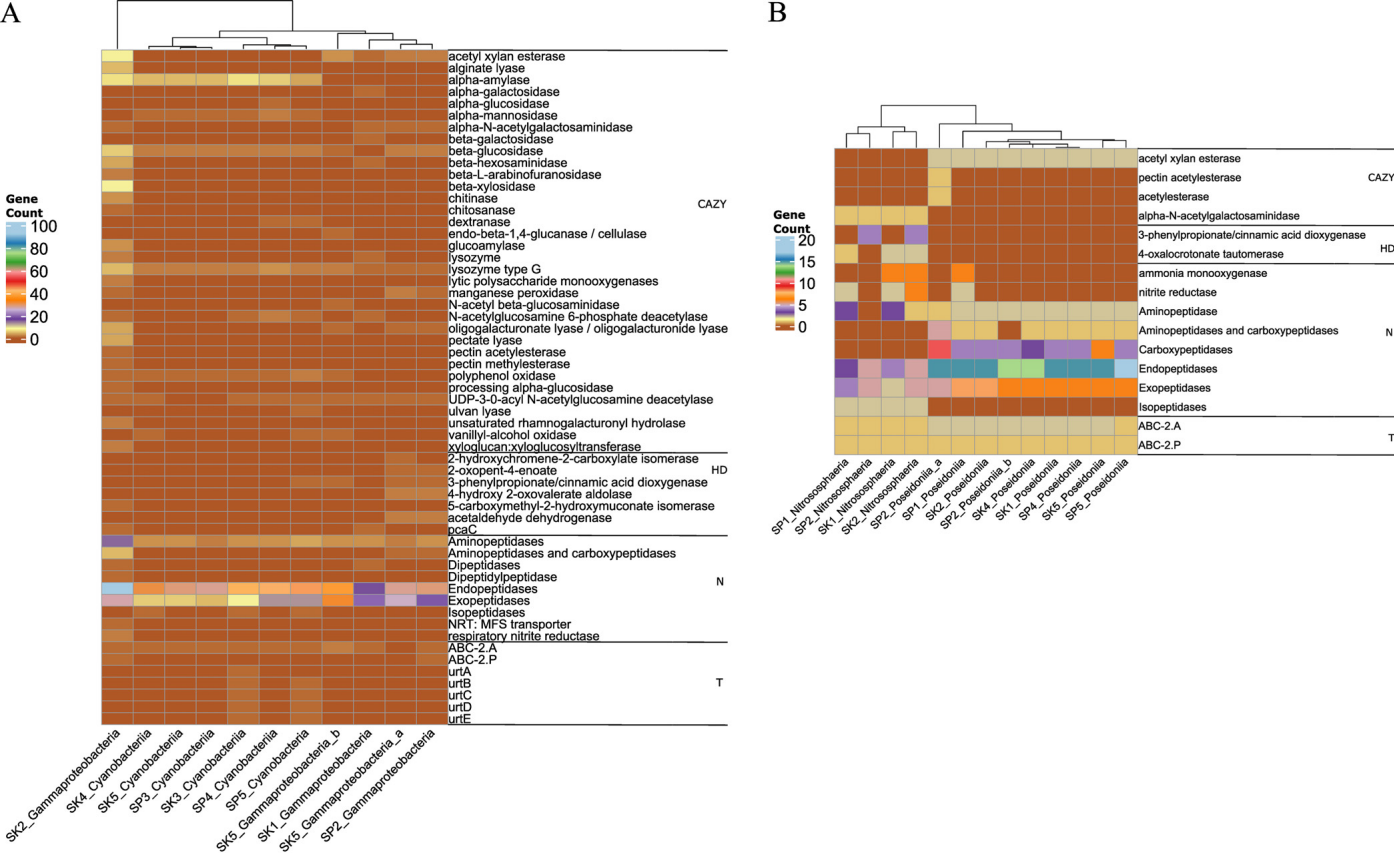
Predicted and identified genes from the 24 MAGs involved in carbohydrate-active enzymes (CAZymes [CAZY]), hydrocarbon degradation (HD), nitrogen metabolism (N), and transporter systems (T) based on the DRAM pipeline. The color scale represents the gene counts/copies. (A) Bacterial functional annotation; (B) archaeal functional annotation.

The average nucleotide identity (ANI) scores of all 11 bacterial and 13 archaeal MAGs were below 90% against 1,254 bacterial (*Cyanobacteriia* and *Gammaproteobacteria*) (Fig. 4A) and 4,957 archaeal (*Thermoplasmatota* and *Thaumarchaeota*) (Fig. 4B) RefSeq complete genomes, respectively. Furthermore, the genomic potential suggests that bacterial genomes reconstructed from the sinking fraction had more CAZymes involved in the degradation of carbohydrates (acetyl xylan esterase), starch (α-amylase), amorphous cellulose (β-glucosidase), and chitin (lysozyme type G). The analyses revealed that gammaproteobacterial MAGs, from station 2, had more CAZymes (Fig. 5A; Data Set S1, Tab 6). We also found that cyanobacterial MAGs appear to have unique CAZymes, including genes involved in the degradation of α-mannan (α-mannosidase), arabinan (dextranase), and sulf-polysaccharides (ulvan lyase). These CAZymes were mostly retrieved in samples from the suspended fraction. Bacterial genomes from station 2 (mostly suspended fraction) and station 5 (mostly sinking fraction) had genes involved in the degradation of hydrocarbon compounds, including catechol (2-oxopent-4-enoate), naphthalene (2-hydroxychromene-2-carboxylate isomerase), and *trans*-cinnamate (4-hydroxy 2-oxovalerate aldolase). On the other hand, *Poseidoniia* harbored CAZymes involved in the degradation of carbohydrates (acetyl xylan esterase). Only one genome (SP2_Poseidoniia_a) had CAZyme genes, including those coding for pectin acetylesterase and acetylesterase (Fig. 5B; Data Set S1, Tab 7), whereas, *Nitrososphaeria* MAGs had CAZyme genes and hydrocarbon degradation genes involved in the degradation of mucin (α-*N*-acetylgalactosaminidase), catechol (4-oxalocrotonate tautomerase), and *trans*-cinnamate (3-phenylpropionate/cinnamic acid dioxygenase). These were mostly recovered from the station 1 and 2 sinking fractions, respectively.

A *Gammaproteobacteria* genome affiliated with *Alteromonadaceae*, from the sinking fraction of station 2, had a respiratory nitrite reductase gene. This gene plays a key role in dissimilatory nitrate reduction. However, several bacterial MAGs had genes involved in the degradation of proteins to amino acids using aminopeptidases and endo- and exopeptidases. All archaeal MAGs had genes involved in the degradation of peptides, including amino-, carboxy-, endo-, and exopeptidases. Samples from stations 1 and 2 (both fractions) had nitrite reductase genes involved in the denitrification process and also had ammonia monooxygenase genes implicated in nitrification—mostly in the sinking fraction. Evidence suggests that these bacterial and archaeal genomes may be chemoautotrophs (Text S1). All bacterial and the archaeal genome (*Poseidoniia*) had genes implicated in photorespiration via the glycine cleavage system, whereas only *Cyanobacteria* MAGs were photoautotrophs with genes related to photosynthesis (psa A-F and psb A-F genes) (Fig. S4; Data Set S1, Tabs 6 and 7). On the other hand, all bacterial and archaeal genomes had the ABC-2.A transporter system (Fig. 5; Data Set S1, Tabs 6 and 7). Additionally, gammaproteobacterial genomes from station 2 (suspended and sinking fractions) and all archaeal MAGs had the ABC-2.P transporter system (Fig. 5; Data Set S1, Tabs 6 and 7). Only *Cyanobacterial* MAGs, from the station 3 sinking fraction and station 5 suspended fraction, had genes implicated in the urea transport system (*urt*A-E) (Fig. 5A; Data Set S1, Tab 6).

10.1128/msphere.00420-22.4 FIG S3 Taxonomic classification of the prokaryotic community from unbinned contigs at the phylum level. The color scale represents read abundance. (A) Bacterial community; (B) archaeal community presented as relative abundance. Download FIG S3, TIF file, 0.9 MB. Copyright © 2023 Dithugoe et al. 2023 Dithugoe et al. https://creativecommons.org/licenses/by/4.0/ This content is distributed under the terms of the Creative Commons Attribution 4.0 International license.

10.1128/msphere.00420-22.5 FIG S4 Predicted genes involved in carbon utilization and chemoautotrophic genomic potential. The color scale represents gene counts/copies. (A) Bacterial central metabolism and electron transport chain (ETC) complexes; (B) Archaeal central metabolism and ETC complexes. Download FIG S4, TIF file, 1.7 MB. Copyright © 2023 Dithugoe et al. 2023 Dithugoe et al. https://creativecommons.org/licenses/by/4.0/ This content is distributed under the terms of the Creative Commons Attribution 4.0 International license.

## DISCUSSION

Organic carbon and nitrogen are the main resource supporting diverse microbial communities in both suspended and sinking particle-pools (42). The microbial genomic potential subsequently alters the nature of both the particulate and dissolved organic pools. Microbial communities, therefore, contribute to the quantity and quality of organic carbon/nitrogen that is effectively exported below the seasonal mixed layer. Knowledge of prokaryotic community composition and their genomic potential on both sinking and suspended particles is thus required to facilitate a mechanistic understanding of prokaryotic contributions to POC/particulate organic nitrogen (PON) degradation/synthesis, which impacts the efficiency of the BCP. While limited to 24 MAGs, this study addresses this knowledge gap by providing the first metagenome-assembled genomes from suspended and sinking particle-pools in the Southern Ocean. The limited number of stations does, however, constrain our ability to interpret the complete functional potential within the context of POC/PON flux variability at the SOTS.

### Differences in prokaryotes may explain divergence in POC and PON contents of sinking and suspended particle-pools.

There is a complex interplay among several factors (e.g., particle composition, sinking rates and prokaryotic activity, etc.) that influences the flux of POM from the surface layer (47, 48). For example, recent studies have demonstrated a positive relationship between phytoplankton biomass and the magnitude of POM export (49, 50), with our data corroborating these findings. In addition, POM content (labile, semi-labile, recalcitrant, or refractory) rather than concentration is considered the main driver of prokaryotic community structure (51, 52), with sinking POM subject to degradation by several FL and particle-attached (PA) prokaryotes (44) that alter its chemical and biological properties (53). Prokaryotic communities (i.e., PA and FL) may in turn contribute as secondary drivers of change, by altering their community structure and/or associated function in response to the altered POM (38, 54, 55). For example, prokaryotes can degrade polysaccharides from POM into labile or semi-labile DOM, while also producing RDOM or rDOM (30, 34). The RDOM compounds act as “sticky polysaccharides,” which then aggregate into POM (28, 30, 56).

Since sinking POC flux is insufficient to meet the carbon demands of prokaryotes, suspended particles are considered a major sustaining source of organic carbon for microbes in the mesopelagic (44). While the concentration of POC in sinking particles decreases exponentially with depth, the concomitant POC concentration in suspended particles remains largely constant and is typically ~1 to 2 orders of magnitude higher than that of sinking particles (44). Our data support these findings, with substantially higher POC concentrations in the suspended than the sinking fraction. This was, however, not true for PON, which typically had more PON (or similar concentrations) in the sinking material than in the suspended material.

Several factors may account for variability in POC and PON concentration, which alters POC/PON ratios below or above the Redfield ratio (6.6) (57). Factors that increase POC/PON ratios include (i) preferential degradation of nitrogen-rich POM, (ii) synthesis of refractory POC resistant to further degradation, and (iii) chemoautotrophic microbial activity on POM, including exopolymer substances (EPSs). Whereas factors that decrease POC/PON include (i) the presence of PA diazotrophs (N_2_-fixing prokaryotes) (58, 59), (ii) oxidation of sinking POC that drives preferential reduction in POC relative to PON, and (iii) nutrient limitation (60). Despite large differences in the distribution of POC/PON ratios between suspended and sinking samples and between stations, the bacterial community compositions were very similar. This similarity at the class level suggests that variability in POC/PON ratios may be influenced by prokaryotic functional potential. On the other hand, differences were observed in archaeal communities at the class level, such that PON contents might have been different between stations. Alternatively, the compositions of source material may have been similar, while degradation by bacteria and archaea may have driven secondary changes in their community structure at the family level (25, 38, 61). There is evidence for this argument in the abundance of bacteria from the *Proteobacteria* and *Bacteroidota* phyla, which suggests variability in bacterial potential between suspended and sinking pools, and between stations, despite similarities in community composition at the class level. As such, differences in genomic potential rather than diversity, particularly in the case of bacteria, might impact the signature of POC and PON (62–64) in both sinking and suspended material at the class level. Nevertheless, the impact of archaeal diversity on POC/PON variability is evident when observing *Nitrososphaeria* (*Nitrosopumilaceae*), which were highest at stations with the highest POC/PON ratios and POC flux. This is likely due to *Nitrososphaeria* having more genes coding for peptidases that degrade polypeptides (reducing PON concentration) releasing ammonia, which was further oxidized to nitrous oxide nitrification (nitrite reductase) and denitrification (ammonia monooxygenase) processes. Conversely, station 3 had the lowest abundance of *Nitrososphaeria*, coincident with particularly high PON flux relative to other stations.

### Prokaryotic genomic potential based on POC and PON content.

Despite extensive studies that focus on prokaryotes from sinking particles from sediment traps (14, 65–67), few focus on prokaryotes from suspended and sinking particles collected from an MSC (42, 44). Based on amplicon sequencing data, M. T. Duret et al. (44) reported niche differentiation between sinking and suspended pools below the epipelagic zone (44). However, prokaryotes may detach from sinking particles, thereby enriching the suspended particle pool with microbes that are similar to those found on sinking particles (42). Our data suggest clear functional differences in PA and FL prokaryotes associated with suspended and sinking POM. For instance, *Proteobacteria* (*Gammaproteobacteria*) MAGs and *Bacteroidota* unbinned contigs had the genomic potential to degrade polysaccharides produced by phytoplankton with specific CAZymes, which is in line with previous findings (68), while archaeal functional predictions suggest capacity to degrade proteins, secondary metabolites, and carbohydrates from POM, also in agreement with previous reports (69). Therefore, despite the phylogenetic similarities between our MAGs, there is evidence that prokaryotes exhibit diverse functional traits. Most specifically, we found higher potential for degrading complex organic carbon in the sinking fraction, consistent with previous reports (67). In addition, taxonomic classification from unbinned contigs and MAGs suggests that the bacterial community (e.g., *Proteobacteria*) may be more prevalent in the sinking particle pool, with more genes involved in the degradation of complex POM. This particular finding is in contrast to previous studies, which have instead shown these degradation genes to be more abundant in the suspended fraction (44). A. O. Leu et al. (67) reported that PA microbes had more diverse CAZymes, extracellular peptidases, and substrate-specific transporters than their FL counterparts. That said, it is likely that any genomic potential to use labile DOM, resulting in the formation of polymers, may consequently initiate aggregation (38), which may subsequently enhance POM export flux, thus accounting for the presence of PA and FL prokaryotes in both sinking and suspended material. Additionally, our results suggests that labile DOM (e.g., glucose) uptake, via the PTS-EIIA transport system (70), might be used in the synthesis of building blocks for key biopolymers (71) that are then exported out of the cell using the ABC2 transport system (72). In such instances, bacterial communities associated with sinking POM may lead to the formation and release of complex organic compounds (i.e., RDOM) from labile DOM (14, 62). *Bacteroidota* contigs and gammaproteobacterial MAGs from this study appear to be more prevalent in the sinking particle pool, which is in agreement with previous reports (14, 73). On the other hand, the sinking POM may be colonized by FL prokaryotes, which use oligosaccharides and other organic compounds released from particle degradation (74, 75).

Prokaryotic genomes and unbinned contigs in both suspended and sinking fractions revealed genomic capacity to fix CO_2_, including phosphoenolpyruvate carboxylase (Arnon-Buchanan cycle), ribulose-bisphosphate carboxylase (Calvin cycle) and acetyl coenzyme A (acetyl-CoA) synthetase (methanogenesis). Prokaryotes that exhibit this genomic potential are typically chemoautotrophs, which synthesize polysaccharide polymers (e.g., RDOM) from CO_2_ (76) to form smaller particles secreted through the ABC2 transport system (72, 77). These small particles are produced at depth via RDOM aggregation or the disaggregation of larger particles (20) and may form part of the particle injection pump through physical processes (18, 19). In addition to fixing CO_2_, the members of the PA chemoautotrophic bacterial community resemble FL bacteria; however, this is not surprising since bacteria constantly attach or detach from particles enriching either the particle or suspended pool with chemoautotrophic taxa that degrade high-molecular-weight compounds (42, 78).

Indeed, there has been some debate regarding the similarity of FL and PA prokaryotic communities, with some studies suggesting that these taxa may be similar (79, 80), while others suggest stark dissimilarities (81, 82). These characteristics may be affected by many factors, including particle sources and substrate availability (83), particle residence times, geographic location (84), and nutrient gradients (85). Here, we collected metagenomic samples in both suspended and sinking fractions to elucidate both FL and PA prokaryotic contributions to BCP efficiency. Communities from suspended and sinking particles showed some evidence of chemoautotrophic potential, which is consistent with previous findings on FL prokaryotes (86). These chemoautotrophic communities also have the genetic capacity to produce labile DOC, thereby sustaining the microbial food web when organic carbon content in the water column is mostly rDOM (87, 88). The chemoautotrophic responses to carbon sources (either inorganic or organic carbon), due to environmental and biological stimuli, nonetheless remain unclear, with ongoing studies needed to investigate the fate of the POM produced by chemoautotrophs (87).

There was also a discernible difference in genomic potentials between prokaryotic communities from MAGs associated with suspended material and those associated with sinking material (notably at station 2). For instance, bacteria from the *Bacteroidetes* and *Proteobacteria* phyla (*Gammaproteobacteria* MAGs) were present in both sinking and suspended samples at station 2, and both possessed various CAZyme genes. However, there were more genes involved in the degradation of labile and complex POM found in the sinking pool. In addition to the gene copies being higher, we found several genes implicated in diatom-derived POM (89, 90), grass POM, and virus-induced POC from picocyanobacterial and polysaccharides (91) in the sinking pool. It is expected that these mechanisms may result in reduced POC flux via particle degradation while sinking into the mesopelagic. All bacterial communities from both MAGs and unbinned contigs were associated with the degradation of chitin, regardless of their association with suspended or sinking material. Chitin is rich in both carbon and nitrogen that can be reintegrated into biomass-forming polysaccharide polymers or remineralized to enrich the water column (92), thus reducing the export flux of both POC and PON.

In addition, the taxonomic classification of prokaryotes and genomic potential (on either the suspended or sinking fraction) was insufficient to infer preference, due to functional evolution involving gene gain and loss (20, 93). Gene gain and loss may occur through the deletion or insertion of genes, including genomic islands, via nonhomologous recombination mechanisms and mobile genetic elements (94). For instance, the rate of bacterial growth and organic matter consumption of ~0.1 to 0.7 day^−1^ (95, 96) occurs within the settling time of 0.833 day^−1^ for the separation of particles. This may explain the difference observed in genomic potential between closely related taxa (*Cyanobiaceae*), complicating the changes observed in bacterial genomic potential in suspended and sinking fractions (14, 26).

Our chemoautotrophic archaeal MAGs from Marine Group I (e.g., *Nitrososphaeria*), specifically those associated with *Nitrosopumilaceae*, were prevalent at stations 1 and 2 while virtually absent in both suspended and sinking fractions at stations 4 and 5. Chemoautotrophic archaeal MAGs are predicted to have genes involved in the degradation of hydrocarbons (4-oxalocrotonate tautomerase), notably in the sinking particle pool (97), which was indeed the case for our samples at stations 1 and 2. This was in-line with predicted archaeal MAG distribution known to be prevalent in the sinking particle-pool scavenging complex POM (26, 67, 98). Previous studies have also shown that archaeal communities may colonize and degrade complex POM (99, 100), with their genomic potential thus more commonly appearing in the sinking particle-pool. Marine Group II (e.g., *Poseidoniia*), associated with *Thalassoarchaeaceae*, are also predicted to play a major role in PON transformations based on the protein degradation pathways recovered (100, 101). These heterotrophs typically use low-molecular-weight PON (102) and, in this study, were more dominant in the suspended particle pool. Our results suggest that *Poseidoniia* use peptidases to degrade protein to amino acids, while *Nitrososphaeria* utilize ammonia released from the degradation of amino acids, which are subsequently converted to nitrous oxide and removed from the system, thereby reducing PON flux at stations 1 and 2. There is, however, no direct evidence (e.g., mechanistic experiments or metatranscriptomic data) of the role of archaea in utilizing PON (e.g., protein) and interactions with RDOC in the ocean.

Ammonia-oxidizing archaeal (AOA) MAGs with the ammonia monooxygenase gene were present in the sinking fraction, at stations 1 and 2 (*Nitrososphaeria*), and the suspended particles, at station 1 (*Poseidoniia*). The AOA have the capacity to use labile and complex organic nitrogen as their main source of ammonia and nitrite and may therefore influence the POC/PON ratio (103). These AOA harbor peptidases that enable the degradation of nitrogen-rich POM, resulting in low concentrations of PON relative to POC in the suspended fraction. Several studies have shown that members of Marine Group I typically dominate the particle-associated AOA community (104) and are involved in the uptake and assimilation of ammonia (98) and the release of DON via the degradation of particles (105, 106). However, *Proteobacteria* contigs and MAGs from the sinking samples at station 2 had the genomic potential for dissimilatory nitrate reduction via respiratory nitrite reductase. Interestingly, this pathway was also found at the sinking particle fraction at station 5, from unbinned *Proteobacteria* contigs. *Proteobacteria* from unbinned contigs and gammaproteobacterial MAGs indicate potential genetic capacity for decreasing nitrogen-rich POM, thus potentially driving an elevated POC/PON ratio. Nitrite reduction (nitrite reductase) was also more prevalent in archaeal than bacterial MAGs. The presence of AOA (*Nitrososphaeria* and *Poseidoniia*), nitrite-oxidizing bacteria (NOB) (*Gammaproteobacteria*), and nitrite-oxidizing archaea (NOA) (*Nitrososphaeria*) MAGs at station 1 (suspended and sinking) and station 2 (suspended) had the highest POC/PON ratio, the highest POC flux, and the lowest PON flux. These NOB/NOA and AOA are obligatory partners where the AOA catalyze the oxidized ammonia released from PON to nitrite and NOB/NOA, which further oxidize the nitrate to nitrate (107), thus possibly explaining the decrease in PON flux at these stations. NOB/NOA and AOA are also key players in the removal of nitrogen from PON, increasing the POC/PON ratio at stations 1 and 2 and subsequently increasing POC export flux relative to PON. In addition to preferential degradation of PON, archaeal MAGs may also be involved in the synthesis and secretion of RDOC via ABC2.A and P transport systems, enriching the water column with organic carbon particles, thus increasing POC relative to PON flux (103).

Although *Cyanobacteria* (*Cyanobiaceae*) are well-known photosynthetic microbes involved in nitrogen fixation (45, 108), our unbinned contigs and MAGs showed no evidence for nitrogen fixation, with no diazotrophic *Cyanobacteria*. This is not unexpected as many *Cyanobiaceae* (*Synechococcus*) do not have genes for nitrogen fixation (109–111). Nonetheless, noncyanobacterial diazotrophs (NCDs), such as dinitrogen (N_2_)-fixing bacteria and archaea, might be present (112). Indeed, *Gammaproteobacteria* (on the sinking sample at station 2) were the only bacterial MAG containing nitrogen metabolism, while *Nitrososphaeria* were also present at stations 1 and 2. The *Poseidoniia* MAG at station 1 (suspended) also had the genomic potential for nitrogen metabolism. Coincidentally, these were the two stations with the highest POC/PON ratio (and highest carbon flux), indicative of preferential nitrogen uptake by the prokaryotic community. Since phytoplankton biomass accounts for only ~6% of the nitrogen flux, the high PON flux observed at stations 3 to 5 may be due to prokaryotic activity on PON by assimilating the available inorganic nitrogen into its biomass (113). The dissimilation of inorganic nitrogen, from PON (114), favors PON export and is thus more likely to be a result of prokaryotic activity.

### Conclusion.

Particulate organic matter (POM) signatures in suspended and sinking particles are influenced by both bacterial and archaeal communities as PA and FL, respectively. Data from genomic potential suggest that the variability in archaeal communities can impact PON flux, whereas the bacterial communities may impact POC flux. Archaeal MAGs were consistent with the predicted dominance of taxa known to scavenge PON in the suspended particle-pool. The relationship between phytoplankton and prokaryotes in POM export is complex. Mechanistic studies may shed light regarding the precise mechanisms governing the relationship between phytoplankton and prokaryotes. These studies may provide further insights into trophic interactions between phytoplankton and prokaryotes and their respective contribution to POM export.

## MATERIALS AND METHODS

### Site description and Marine Snow Catcher sample collection.

The Southern Ocean Time Series (SOTS) site is located at 47°S and 142°E, ~530 km southwest of Tasmania, in the Indian/Australian sector of the SAZ (115). Samples were collected from five Marine Snow Catcher (MSC) stations (Fig. 1A) over the course of 2 weeks between March and April 2019 aboard the RV *Investigator*. The Marine Snow Catcher (MSC) was deployed at 10 m below the mixed-layer depth (MLD) (see Data Set S1, Tab 1, in the supplemental material) following the method described by J. S. Riley et al. (12) (see Text S1 in the supplemental material). The POC/PON samples collected from the MSC were analyzed in the Department of Archaeology at the University of Cape Town. The POC/PON concentration determination and flux calculations were adjusted based on J. S. Riley et al. (12) (Text S1).

### Molecular analysis and sequencing.

To explore the composition and function of the entire prokaryotic community associated with the suspended and sinking particles, including the FL community, 2 L of water from both fractions was filtered using 0.2-μm-pore-size polycarbonate membrane filters (47-mm diameter); Millipore (Burlington, MA, USA) (115). The filters were stored at −80°C until further processing. DNA was extracted using the PowerSoil kit (Qiagen, Hilden, Germany) as described by M. Hirai et al. (116). The resultant DNA was sequenced by Admera Health Biopharma Services (South Plainfield, NJ, USA) (Text S1).

### Taxonomic classification and MAG reconstruction.

The quality of raw metagenomic data was assessed using FastQC (https://github.com/s-andrews/FastQC). The reads were processed to remove sequencing adapters and low-quality reads using Trimmomatic v.0.36 (117). These reads were used for taxonomic classification, using the default parameters in SingleM v.0.13.2, concentrating on 14 single-copy marker genes, ribosomal protein genes for differentiation of species (https://github.com/wwood/singlem). The ATLAS workflow (118) was used to assemble raw reads and for generating MAGs using the default parameter settings. Unbinned metagenomic contigs with lengths of ≥2.5 kb were assigned taxonomy using Contig Annotation Tool (CAT) with the default settings (119). The top phyla from CAT output were extracted and concatenated for DRAM annotation. CheckM v.1.1.3 was used to assess the quality of MAGs as detailed previously (120). Following genome reporting standards, MAGs with genome completeness scores of >50% and <10% contamination were selected for downstream analysis (121). About 24 of our MAGs were medium-quality drafts with ≥50% completeness and <10% contamination. The Genome Taxonomy Database toolkit (GTDB-Tk) v.1.5.0 was used to assign taxonomy to all MAGs. Phylogenetic diversity of the reconstructed MAGs was inferred against complete archaeal and bacterial genomes acquired from the NCBI RefSeq database using the FastANI v.1.32 tool (122). To estimate the abundance of each taxon, MAGs from the suspended and sinking particle-pools were mapped using default parameters in CoverM v.0.6.1 (https://github.com/wwood/CoverM). Gene calling of the unbinned metagenomic contigs retrieved from CAT and the MAGs was obtained by using DRAM v.1.2.0 with 6 databases, including UniRef90, Pfam, dbCAN, RefSeq viral, VOGDB, and MEROPS, with E value of <10^−15^ (123).

### Data availability.

All ten raw metagenomic sequences collected from the Southern Ocean Time Series stations are available at the NCBI (https://www.ncbi.nlm.nih.gov/bioproject/PRJNA749920).
