# Supplementary figures and images for: Bacteria and Archaea Regulate Particulate Organic Matter Export in Suspended and Sinking Marine Particle Fractions
Source: mSphere. 2023 Apr 24;8(3):e00420-22. doi: 10.1128/msphere.00420-22 (PMC10286711; doi:10.1128/msphere.00420-22)

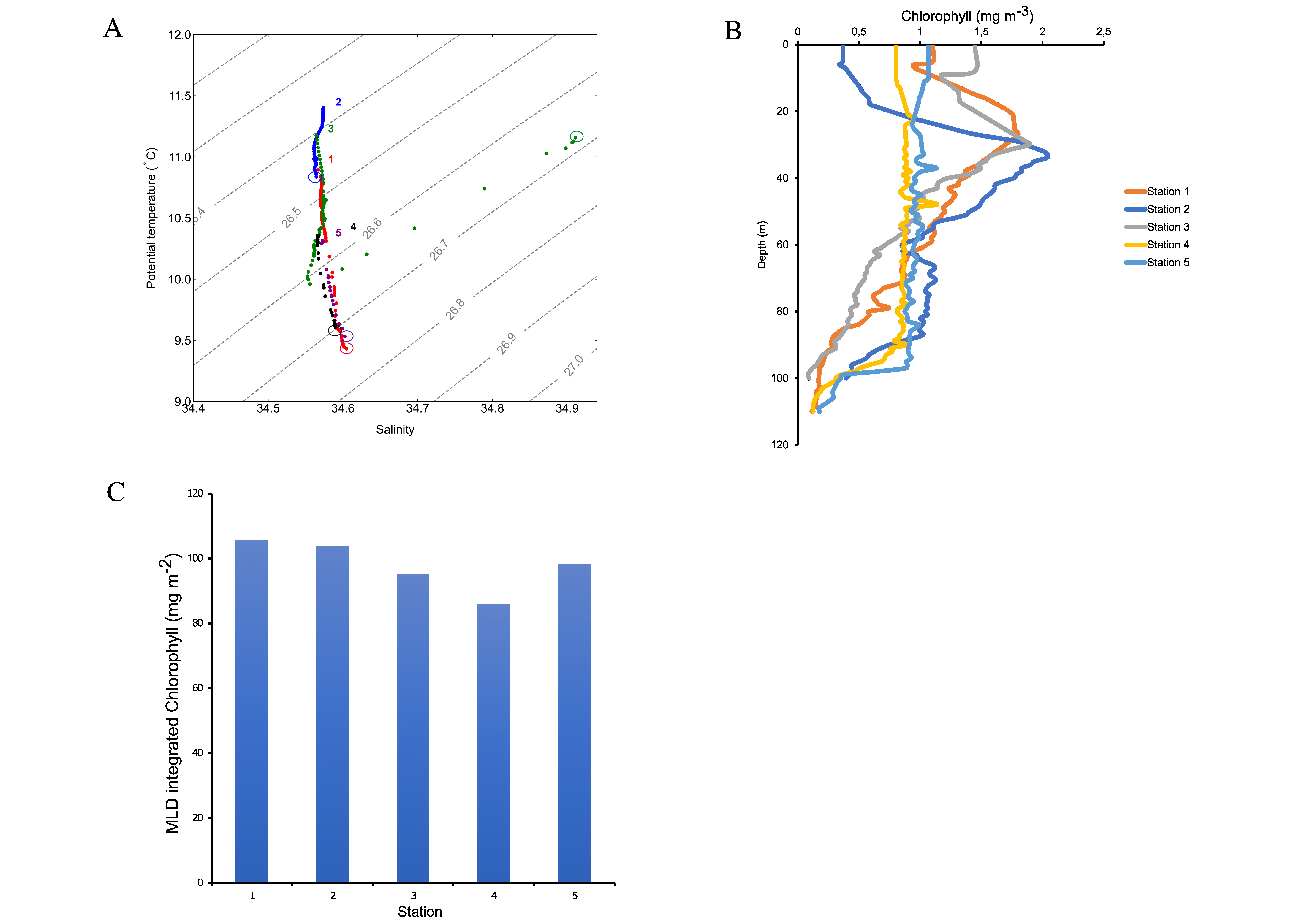

Supplement: FIG S1 [file msphere.00420-22-s0001.tif]

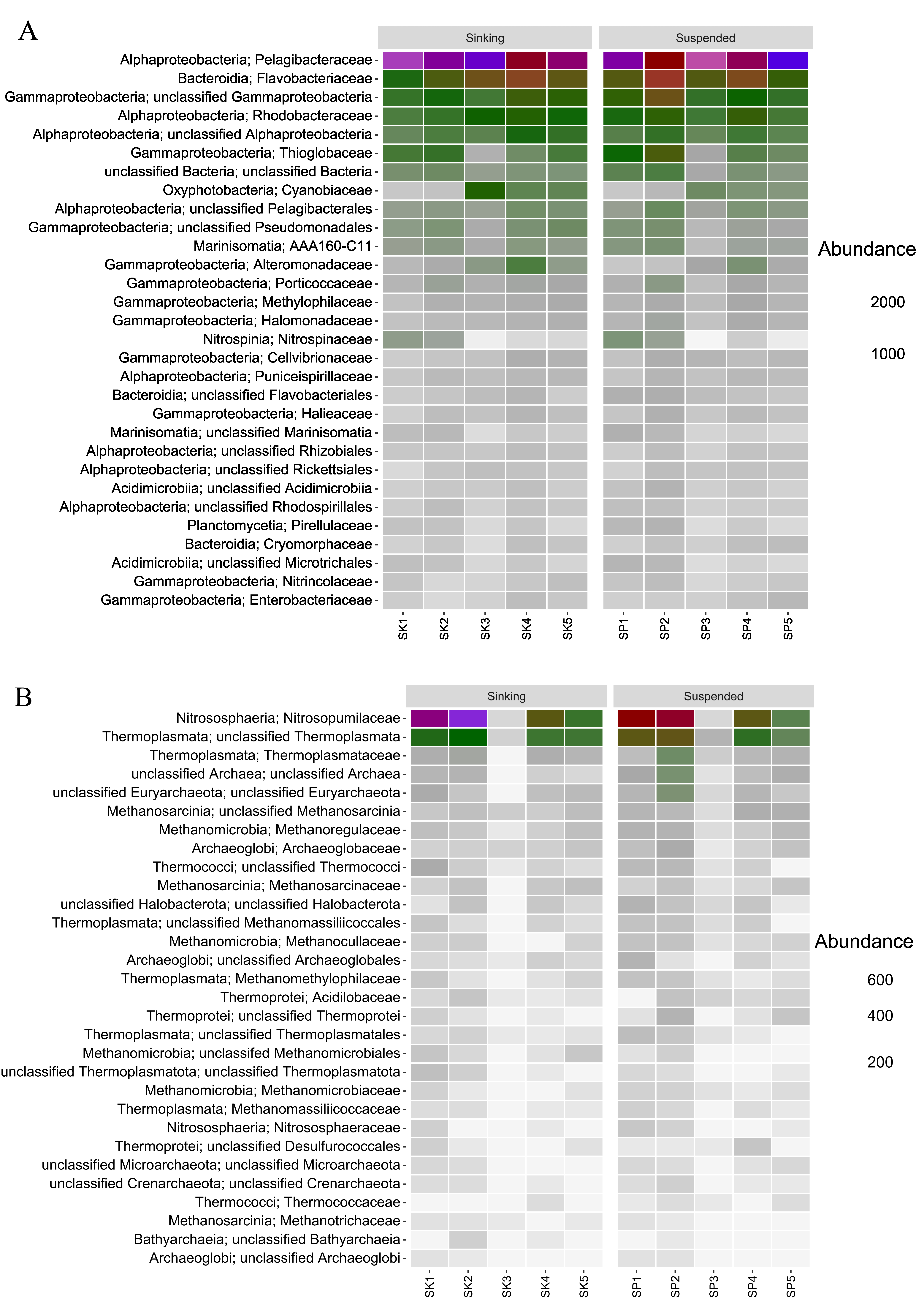

Supplement: FIG S2 [file msphere.00420-22-s0002.tif]

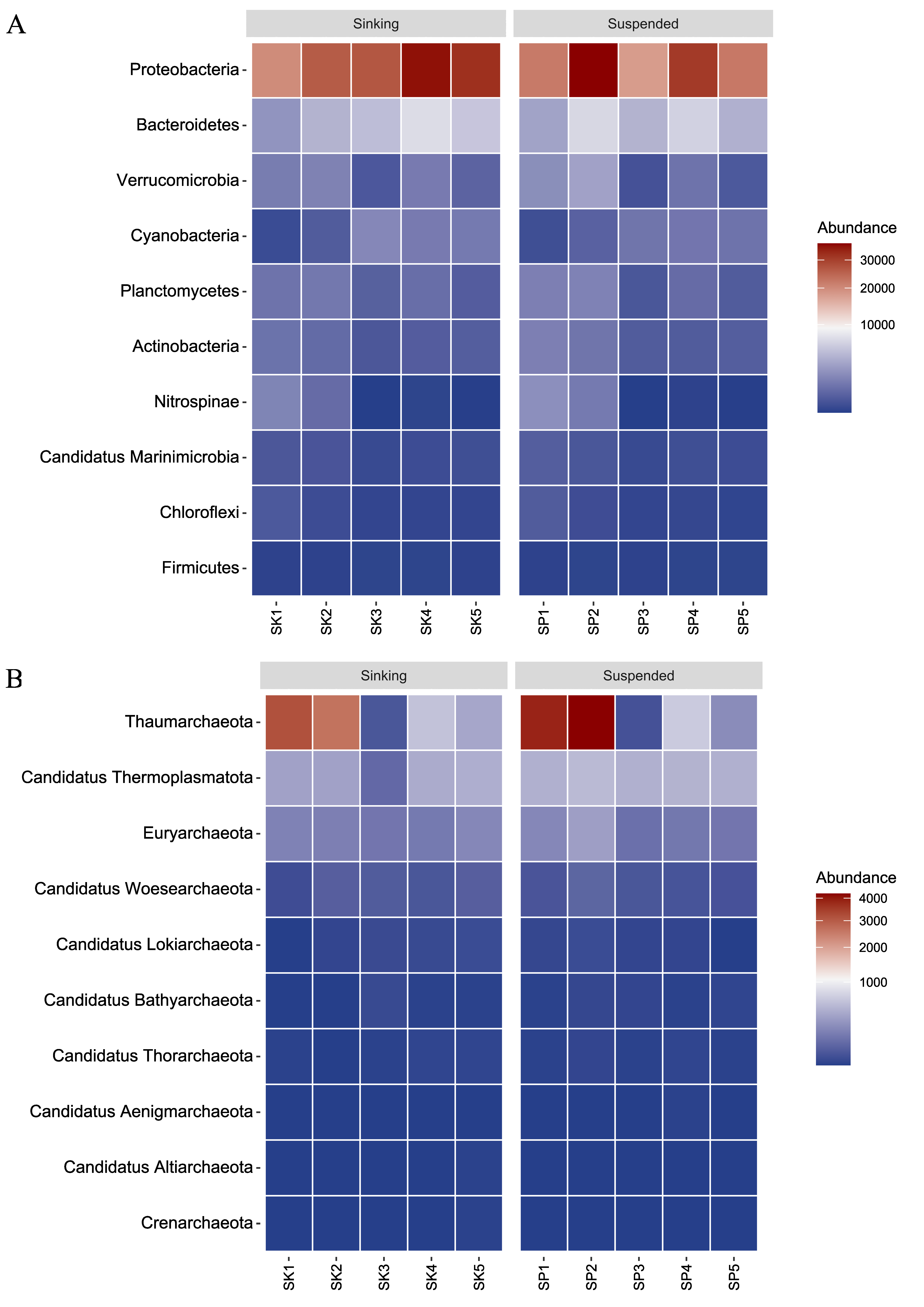

Supplement: FIG S3 [file msphere.00420-22-s0003.tif]

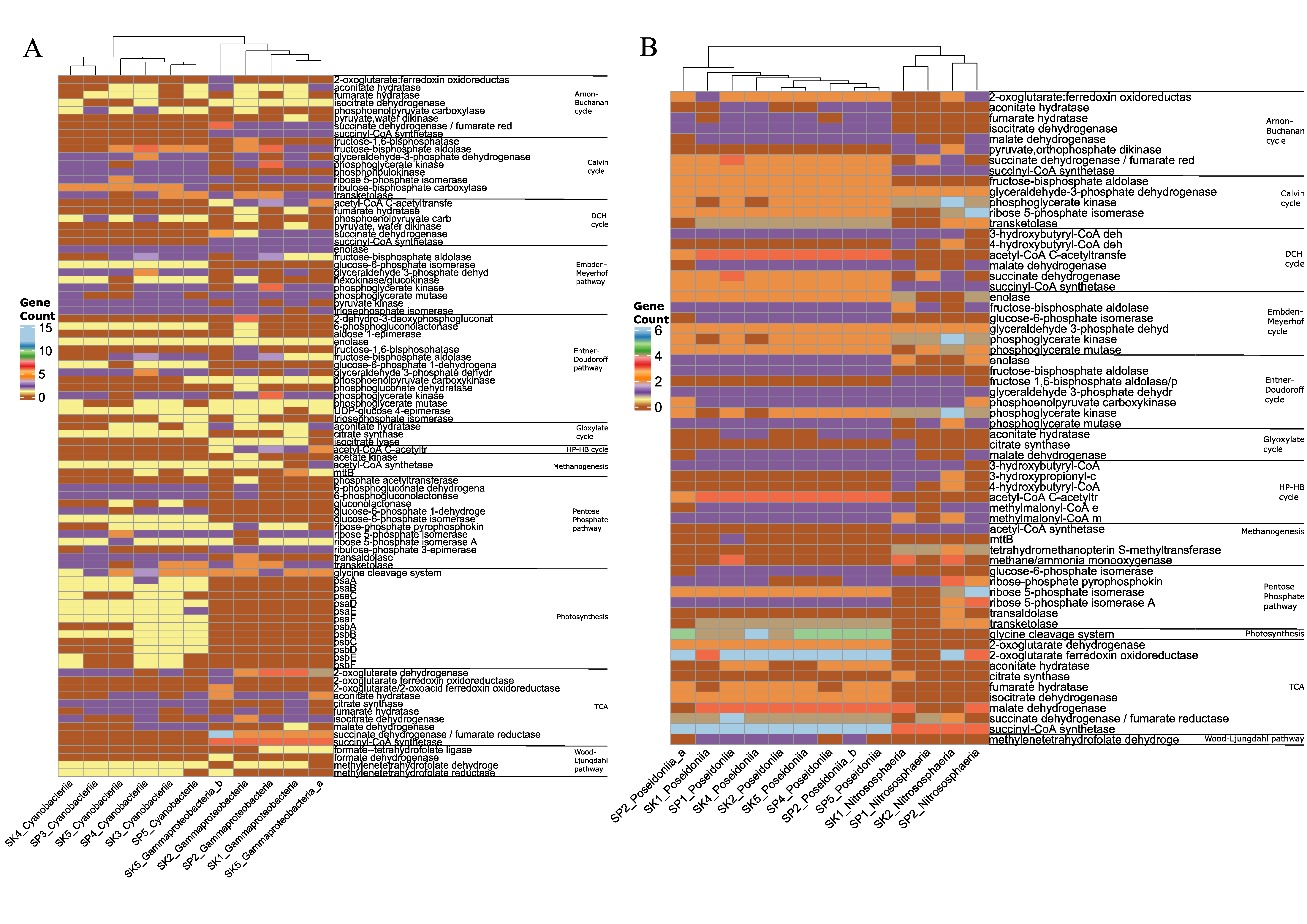

Supplement: FIG S4 [file msphere.00420-22-s0004.tif]
