# Supplementary material for: Bacteria and Archaea Regulate Particulate Organic Matter Export in Suspended and Sinking Marine Particle Fractions
Source: mSphere. 2023 Apr 24;8(3):e00420-22. doi: 10.1128/msphere.00420-22 (PMC10286711; doi:10.1128/msphere.00420-22)
Supplement: TEXT S1 [file msphere.00420-22-s0006.docx]

Supplementary text file for Dithugoe et. al .

**Bacteria and Archaea regulate particulate organic matter export in suspended and sinking marine particle fractions**

Table of Contents

[1. Supplementary Materials and Methods 2](#_Toc130387425)

[1.1 Site description and sample collection 2](#_Toc130387426)

[1.2 Marine Snow Catcher sample collection 2](#_Toc130387427)

[1.3 Molecular analysis and sequencing 3](#_Toc130387428)

[2. Supplementary Results 4](#_Toc130387429)

[2.1 Ancillary station data 4](#_Toc130387430)

[2.2 Variations in POC and PON in the suspended and sinking particle-pools 4](#_Toc130387431)

[2.3 Taxonomic profiles and functional annotation of unbinned metagenomic contigs 5](#_Toc130387432)

[2.4 MAG of reconstruction and taxonomic profiling 7](#_Toc130387433)

[2.5 Functional profiling of bacterial and archaeal genomes from the suspended and sinking particle-pools 8](#_Toc130387434)

[3. References 9](#_Toc130387435)

# Supplementary Materials and Methods

## 1.1 Site description and sample collection

The Southern Ocean Time Series (SOTS) is Australia’s contribution to the international Ocean SITES global network of time series observatories (<http://www.oceansites.org/>). The site is located at 47⁰S and 142⁰E, approximately 530 km southwest of Tasmania in the Indian/Australian sector of the SAZ (48). At each station, CTD deployments provided temperature, salinity, and fluorescence derived chlorophyll profiles. The mixed layer depth (MLD) was calculated from temperature profiles with a threshold criterion of 0.2ºC (1).

## 1.2 Marine Snow Catcher sample collection

The Marine Snow Catcher (MSC) was deployed at 10 m below the MLD (Supplementary Table 1) following the method described by J. S. Riley et al. (2). A MSC acts as a settling chamber allowing the separation of suspended (top of the MSC) and sinking (bottom of the MSC) samples after 2 hours of settling. The approach of the MSC is to allow suspended and sinking particles to separate by their sinking velocity and as such, particles with low sinking rates may be missed (3). After sampling, both the suspended and sinking particle-pools were homogenized and divided using a Folsom splitter for POC, particulate organic nitrogen (PON) and molecular analysis (without replicates due to volume constraints). Sub-samples for POC/PON analysis were filtered onto pre-combusted (450⁰C, 12 h) glass fibre filters (47 mm diameter GF/F, Whatman) using a vacuum filtration pump at a pressure of -0.2 bar. The filters were then placed in sterile petri-dishes and oven dried overnight at 25⁰C. This was followed by acid fumigation with concentrated hydrochloric acid overnight to remove inorganic carbon. Filters were then punched and folded into aluminium tin cup foils. The samples were analysed in the Department of Archaeology at the University of Cape Town on a Flash 2000 organic elemental analyser (Thermo Fisher Scientific, Waltham, MA, USA). The POC/PON concentrations for the sinking fraction were adjusted based on the assumption that suspended POC/PON was homogenous throughout the MSC before settling (i.e., the concentration of the suspended fraction was subtracted from the sinking fraction) (2). The PON:PON ratio was calculated by dividing the concentration of POC by the concentration of PON in both the suspended and sinking fractions. The POC and PON flux was calculated by dividing the sinking mass (mg) with the MSC area (0.06 m^-2^). The resulting value was then divided by the settling time (0.833 days) and multiplied by the ratio of the sampled sinking volume (0.87) based on the MSC design (4).

## 1.3 Molecular analysis and sequencing

The resultant DNA was assessed using Qubit 4 Fluorometer (Thermo Fisher Scientific, Waltham, MA, USA). High quality DNA (3-36 µg.l^-1^) was sequenced by Admera Health Biopharma Services (South Plainfield, USA). Library preparation was performed using the Nextera XT DNA Library Preparation Kit (Illumina, California, USA) as recommended by the manufacturer. The library was quantified using the KAPA SYBR® FAST qPCR with Quant Studio ® 5 System (Applied Biosystems). The libraries were pooled (equimolar concentrations) and sequenced using an Illumina® HiSeq (Illumina, California, USA) using the 2x150 paired end chemistry.

# Supplementary Results

## 2.1 Ancillary station data

Temperature and salinity (TS) plots for all 5 stations (Supplementary Figure S1A) showed very similar water mass characteristics in the surface 120 m, above the MSC sampling depth (Figure 1A). Station 1, 4 and 5 at a sampling depth of 110 m had very similar water mass characteristics, while those stations sampled at 100 m were slightly warmer (station 2) and both warmer and saltier in the case of station and 3 (Supplementary Figure S1A). Station 2 demonstrated slight deviations with increased salinity levels relative to temperature, but only at depths greater than 100 m. As such, any observed variability between the 5 MSC samples was unlikely the result of lateral advection but more likely a reflection of temporal adjustments in the system over the two-week sampling period. MLD’s for all stations were similar and ranged from 100 - 110 m (Data Set S1 Tab 1). Chlorophyll profiles, at stations 1 to 3, exhibited a subsurface maximum at ~30 m indicative of surface nutrient limitation. Stations 4 and 5 were homogenous throughout the mixed layer (Supplementary Figure S1B). MLD integrated chlorophyll (as a proxy for phytoplankton biomass) was highest at stations 1 and 2 (106 and 104 mg m^-2^ respectively) and lowest at station 4 (86 mg m^-2^) (Supplementary Figure S1C).

## 2.2 Variations in POC and PON in the suspended and sinking particle-pools

Despite relatively similar water mass and phytoplankton biomass characteristics between the five stations, differences in POC and PON flux and distribution (suspended versus sinking) between stations were substantial. The large majority of POC was observed in the suspended particle pool (84% ±11%) with only a small percentage found in the sinking pool (16% ±11%) (Figure 1B). PON levels demonstrated the opposite trend with the highest values observed in the sinking pool (68% ±14%) compared to the suspended pool (32% ±14%) (Figure 1C). The distribution of PON between stations was also different, with the highest levels (in both the suspended and sinking fractions) occurring at station 3 (87 and 29 µg l^-1^, respectively) compared to all other stations (< 20 µg l^-1^) (Figure 1D), which drove the highest PON flux at station 3 (31 mg m^-2^ d^-1^) (Figure 1E). For POC, the highest concentrations for sinking pools were found at station 2 (96 µg l^-1^), which consequently had the highest POC flux (34 mg m^-2^ d^-1^); an order of magnitude higher than the POC flux observed at station 4 (3 mg m^-2^ d^-1^). In contrast, suspended POC was highest at stations 2 and 3 (222 and 252 µg l^-1^, respectively). This variability drove large differences in suspended and sinking POC:PON ratios, which were particularly high in the suspended fraction, at stations 1 and 2 (31 and 40 POC:PON, respectively) compared to all other stations (<9). In the sinking fraction, the highest ratios were similarly found at stations 1 and 2 (2 and 6 POC:PON, respectively) compared to all other stations (<1). Positive relationship between MLD integrated chlorophyll and POM flux were observed. However, this relationship was poor (r^2^ = 0.33 for POC and r^2^ = 0.06 for PON), suggesting that phytoplankton biomass may only account for approximately 30% of carbon flux variability and as little as roughly 6% of nitrogen flux variability during austral autumn (post bloom period).

## 2.3 Taxonomic profiles and functional annotation of unbinned metagenomic contigs

Taxonomic assignments, of the unbinned metagenomic contigs, indicated that all samples were dominated mostly by bacterial phyla associated with *Proteobacteria* and *Bacteroidota*. The third most dominant bacterial phyla in station 1, 2 and 4 was *Verrucomicrobia*, whereas samples from station 3 and 5 had *Cyanobacteria* as the third most abundant taxa in both the suspended and sinking particle-pools (Supplementary Figure S3A). On the other hand, archaeal communities in station 1, 2 and 4 were dominated by *Thaumarchaeota*, *Candidatus Thermoplasmatota* and *Euryarchaeota,* respectively (Supplementary Figure S3B). The suspended fraction of station 3 was dominated by *Candidatus Thermoplasmatota*, *Euryarchaeota* and *Thaumarchaeota*, whereas the sinking fraction was dominated by *Euryarchaeota*, *Candidatus Thermoplasmatota* and *Thaumarchaeota*. Station 5 was dominated by *Candidatus Thermoplasmatota*, *Thaumarchaeota* and *Euryarchaeota, respectively*.

Genes were identified for the degradation of hydrocarbon from *Actinobacteria* contigs including naphthalene (2-hydroxychromene-2-carboxylate isomerase), protocatechuate (*pca* C) and trans-cinnamate (3-phenlypropionate/cinnamic acid dioxygenase), with more gene copies in the suspended fraction, mostly at station 2 and 3 (Figure 3A; Data Set S1 Tab 2). Several bacteria were also predicted to have genes involved in carbon utilisation via Embden-Meyerhof pathway (glyceraldehyde-3-phosphate dehydrogenase, phosphoglycerate kinase and pyruvate kinase) and TCA (succinyl-CoA synthetase and succinate dehydrogenase) in both fraction (Data Set S1 Tab 2). Contrary to bacteria, archaea had only a few pathways involved in carbon utilisation, which includes pentose phosphate pathway (ribose 5-phosphate isomerase) and TCA cycle (succinyl-CoA synthetase and succinate dehydrogenase). These pathways appear to be present in both the suspended and sinking fractions (Figure 3B; Data Set S1 Tab 3).

Moreover, bacterial contigs from all stations were predicted to have genes involved in CO_2_ fixation and to produce RDOC via chemoautotrophic pathways. Chemoautotrophic pathways includes 3-hydroxypropionate bicycle (maly-CoA), acetyl-CoA pathway (carbon-monoxide dehydrogenase), Arnon-Buchanan cycle (isocitrate dehydrogenase), Calvin cycle (ribulose-bisphosphate carboxylase and glyceraldehyde-3-phosphate dehydrogenase), Dicarboxylate- and hydroxypropionate-hydroxybutylate cycle (acetyl-CoA C-acetyltransferase), methanogenesis (formylmethanofuran dehydrogenase) and Wood-Ljungdahl pathway (methylenetetrahydrofolate reductase) (Figure 3A; Data Set S1 Tab 2). These pathways, and related gene copies, were higher at station 1 and 2 (suspended), station 3 (both suspended and sinking), and station 4 and 5 (sinking). On the other hand, evidence suggest that the archaeal communities found were chemoautotrophs, based on possessing genes implicated in the Arnon-Buchanan cycle (isocitrate dehydrogenase), Calvin cycle (ribose 5-phosphate isomerase), dicarboxylate- and hydroxypropionate bi-cycle (acetyl-CoA C-acetyltransferase), methanogenesis (acetyl-CoA synthetase) and Wood-Ljungdahl pathway (methylenetetrahydrofolate dehydrogenase) (Figure 3B; Data Set S1 Tab 3).

## 2.4 MAG of reconstruction and taxonomic profiling

In total, 24 medium quality MAGs were recovered (11 from the suspended and 13 from the sinking particle-pool fractions) (Data Set S1 Tab 4). Taxonomic classification revealed that 5 MAGs were affiliated with *Gammaproteobacteria,* with 4 recovered from the sinking particle pool (stations 3 - 5) and only one from the suspended fraction (station 2). In total, 6 MAGs were affiliated with *Cyanobacteriia,* and these were split evenly between the sinking and suspended particle pools from stations 3 to 5. The remaining MAGs were classified as *Poseidoniia* (9 in total occurring in both the suspended and sinking fractions at all stations except station 3) and *Nitrososphaeria* (4 in total occurring in both the suspended and sinking fractions at stations 1 and 2). The Average Nucleotide Identity (ANI) scores of all bacterial and archaeal MAGs were below 90%, against 1254 bacterial (*Cyanobacteriia* and *Gammaproteobacteria*) (Figure 4A) and 4 957 archaeal (*Thermoplasmatota* and *Thaumarchaeota*) (Figure 4B) RefSeq complete genomes, respectively.

## 2.5 Functional profiling of bacterial and archaeal genomes from the suspended and sinking particle-pools

Furthermore, bacterial MAGs had genes involved in carbon utilization via TCA (succinate dehydrogenase) and Embden-Meyerhof pathway (glyceraldehyde 3-phosphate dehydrogenase) (Supplementary Figure S4A). Like bacterial MAGs, archaeal genomes had genes involved in carbon utilization via TCA (succinate dehydrogenase) and were possibly chemoautotrophs based on genes linked to the Calvin cycle (ribose-5-phosphate isomerase) and methanogenesis (acetyl-CoA synthetase) (Supplementary Figure S4B; Data Set S1 Tab 7).

Evidence suggests that these bacterial and archaeal genomes may be chemoautotrophs, based on the possession of several genes involved in the Arnon-Buchanan cycle (phosphoenolpyruvate carboxylase), Calvin cycle (ribulose-bisphosphate carboxylase) and methanogenesis (acetyl-CoA synthetase) (Supplementary Figure S4A, B; Data Set S1 Tab 6, Tab 7).

# References

1. de Boyer Montégut C. 2004. Mixed layer depth over the global ocean: An examination of profile data and a profile-based climatology. Journal of Geophysical Research 109.

2. Riley JS, Sanders R, Marsay C, Le Moigne FAC, Achterberg EP, Poulton AJ. 2012. The relative contribution of fast and slow sinking particles to ocean carbon export. Global Biogeochemical Cycles 26.

3. Puigcorbe V, Ruiz-Gonzalez C, Masque P, Gasol JM. 2020. Sampling Device-Dependence of Prokaryotic Community Structure on Marine Particles: Higher Diversity Recovered by in situ Pumps Than by Oceanographic Bottles. Front Microbiol 11:1645.

4. Fontanez KM, Eppley JM, Samo TJ, Karl DM, DeLong EF. 2015. Microbial community structure and function on sinking particles in the North Pacific Subtropical Gyre. Front Microbiol 6:469.
